# PARP inhibitor veliparib and HDAC inhibitor SAHA synergistically co-target the UHRF1/BRCA1 DNA damage repair complex in prostate cancer cells

**DOI:** 10.1186/s13046-018-0810-7

**Published:** 2018-07-16

**Authors:** Linglong Yin, Youhong Liu, Yuchong Peng, Yongbo Peng, Xiaohui Yu, Yingxue Gao, Bowen Yuan, Qianling Zhu, Tuoyu Cao, Leye He, Zhicheng Gong, Lunquan Sun, Xuegong Fan, Xiong Li

**Affiliations:** 1 Center for Molecular Medicine, Xiangya Hospital, Central South University, 87 Xiangya Road, Changsha, 410008 Hunan China; 2 Hunan Key Laboratory of Molecular Radiation Oncology, Xiangya Hospital, Central South University, Changsha, China; 3 State Key Laboratory of Chemo/Biosensing and Chemometrics, Hunan University, Changsha, China; 4 Research Institute for Prostate Disease, Central South University, Changsha, China; 5 Department of Pharmacy, Xiangya Hospital, Central South University, Changsha, China; 6 Hunan Key Laboratory of Viral Hepatitis, Xiangya Hospital, Central South University, Changsha, China

**Keywords:** PARP inhibitor, HDAC inhibitor, DNA damage, DNA repair, Synergistic effect

## Abstract

**Background:**

The poly ADP ribose polymerase (PARP) inhibitor olaparib has been approved for treating prostate cancer (PCa) with BRCA mutations, and veliparib, another PARP inhibitor, is being tested in clinical trials. However, veliparib only showed a moderate anticancer effect, and combination therapy is required for PCa patients. Histone deacetylase (HDAC) inhibitors have been tested to improve the anticancer efficacy of PARP inhibitors for PCa cells, but the exact mechanisms are still elusive.

**Methods:**

Several types of PCa cells and prostate epithelial cell line RWPE-1 were treated with veliparib or SAHA alone or in combination. Cell viability or clonogenicity was tested with violet crystal assay; cell apoptosis was detected with Annexin V-FITC/PI staining and flow cytometry, and the cleaved PARP was tested with western blot; DNA damage was evaluated by staining the cells with γH2AX antibody, and the DNA damage foci were observed with a fluorescent microscopy, and the level of γH2AX was tested with western blot; the protein levels of UHRF1 and BRCA1 were measured with western blot or cell immunofluorescent staining, and the interaction of UHRF1 and BRCA1 proteins was detected with co-immunoprecipitation when cells were treated with drugs. The antitumor effect of combinational therapy was validated in DU145 xenograft models.

**Results:**

PCa cells showed different sensitivity to veliparib or SAHA. Co-administration of both drugs synergistically decreased cell viability and clonogenicity, and synergistically induced cell apoptosis and DNA damage, while had no detectable toxicity to normal prostate epithelial cells. Mechanistically, veliparib or SAHA alone reduced BRCA1 or UHRF1 protein levels, co-treatment with veliparib and SAHA synergistically reduced BRCA1 protein levels by targeting the UHRF1/BRCA1 protein complex, the depletion of UHRF1 resulted in the degradation of BRCA1 protein, while the elevation of UHRF1 impaired co-treatment-reduced BRCA1 protein levels. Co-administration of both drugs synergistically decreased the growth of xenografts.

**Conclusions:**

Our studies revealed that the synergistic lethality of HDAC and PARP inhibitors resulted from promoting DNA damage and inhibiting HR DNA damage repair pathways, in particular targeting the UHRF1/BRCA1 protein complex. The synergistic lethality of veliparib and SAHA shows great potential for future PCa clinical trials.

**Electronic supplementary material:**

The online version of this article (10.1186/s13046-018-0810-7) contains supplementary material, which is available to authorized users.

## Background

Prostate cancer (PCa) is the most commonly diagnosed malignancy and the second leading cause of cancer death in American males. An estimated 161,360 new cases and 26,730 deaths from PCa were predicted for 2017 [1]. Androgen is crucial for PCa development, and androgen deprivation therapy (ADT) is widely accepted as a first-line treatment for advanced and metastatic PCa [2]. Although ADT is initially effective, most patients eventually progress to metastatic castration-resistant PCa (mCRPC) in 2 to 3 years. Despite the new generation of AR antagonists used alone or in combination with chemotherapeutic drugs for the treatment of mCRPC, mCRPC remains incurable with an average life span less than 19 months [3]. New effective therapeutics is urgently required.

Poly ADP-ribose polymerase (PARP) is involved in the DNA damage response. The PARP inhibitor olaparib was recently approved by the FDA for mCRPC and ovarian cancer patients with *BRCA1/2* or *ATM* gene mutations [4–6]. *BRCA1* and *BRCA2* are two critical tumor suppressor genes crucial for DNA double strand break (DSB) repair through homologous recombination (HR) pathways [7], and play key roles in breast cancer [8, 9]. Approximately 25 to 30% of mCRPC involves somatic mutations of the *BRCA1/2* genes, resulting in DNA repair deficiency [10]. Aberrations of DNA repair genes have been associated with sensitivity to DNA damage drugs such as platinum, radiotherapy and PARP inhibitors [4].

Veliparib is another PARP inhibitor developed by AbbVie USA [11]. The FDA awarded veliparib orphan drug status in November 2016 for non-small cell lung cancer. As of 2017, 96 clinical trials involving veliparib were registered with the FDA based on its anticancer potential in several cancer types. A clinical trial combining abiraterone acetate and prednisone with or without veliparib in patients with metastatic castration-resistant prostate cancer is ongoing (NCT01576172, ClinicalTrials.gov). Limited studies have been performed to directly compare the antitumor efficacy and mechanisms of olaparib and veliparib. It has been reported that oliparib have stronger catalytic inhibitory properties and the potency to trap PARP enzymes to the damage DNA than veliparib [12]. The available data showed that olaparib and veliparib differ in their off-target effects. Olaparib reduced DNA damage repair activity via G2 cell cycle arrest in a p53-dependent manner, but veliparib did not have such an effect [13].

Histone deacetylases (HDACs) play an important role in structural modification and gene expression regulation through induction of histone acetylation. Several HDAC inhibitors have been approved by the FDA to treat hematological malignancies [14, 15]. Although they are not approved by the FDA, HDAC inhibitors have shown the anticancer potential for solid tumors such as PCa in preclinical studies [16, 17]. HDAC1, 2 and 3 are highly expressed and excessively activated in PCa, especially in mCRPC [18]. High expression of HDACs enhances the proliferation and metastatic potential of PCa cells [18, 19], while HDAC inhibitors decrease the potential [20, 21]. Importantly, HDAC is involved in HR DNA repair [22]. HDAC1 and 2 are recruited to DNA break sites when DNA damage occurs, interact with PCNA, and localize to the sites of DNA replication [23, 24]. SAHA, a pan-HDAC inhibitor, prevented DNA damage repair by down-regulating RAD50 and MRE11 [25]. In addition, SAHA and valproic acid, another HDAC1 inhibitor, induced the downregulation of RAD51 expression [26, 27]. Trichostatin A induces a DNA damage signaling pathway in an ATM-dependent manner [28].

Since PARP and HDAC inhibitors prevent HR DNA repair, the combinationof two inhibitors has been tested to improve the anticancer efficacy [29–31]. SAHA significantly improved the anticancer efficacy of olaparib in triple-negative breast cancer (TNBC) cells that expressed functional phosphatase and tensin homolog (PTEN) [32]. By attenuating the levels of DNA damage response and HR proteins ATR, CHK1 and BRCA1, this pan-HDAC inhibitor induces ‘BRCAness’ and sensitizes TNBC cells lacking BRCA1 to the lethal effects of a PARP inhibitor [33]. Additionally, SAHA and olaparib showed synergistic therapeutic effects on prostate cancer cell death, apoptosis and DNA damage by decreasing the protein levels of BRCA1 and RAD51, while have no effect on normal prostate epithelial cells [31]. UHRF1 (Ubiquitin-like with PHD and ring-finger domain 1) is an important protein for DNA methylation maintenance, recognizing specific DNA hemimethylation and recruiting DNMT1 to catalyze methylation on the hemimethylated CpG motifs [34]. UHRF1 is involved in drug resistance and DNA damage repair [35]. UHRF1 recruits ERCC1 and MUS81 to repair DNA lesions [36]. Additionally, when DNA damage occurs, BRCA1 recruits UHRF1 protein to DNA double-strand breaks in S phase, and then UHRF1 mediates K63-linked polyubiquitination of RIF1, resulting in its dissociation from 53BP1 and DSBs, thereby facilitating HR initiation [37]. These findings suggested that UHRF1 plays a critical rolein the combinational therapy of PARP and HDAC inhibitors.

In this study we tested the anticancer efficacy of veliparib and SAHA alone or in combination, using cell viability, colony formation and apoptosis detection assays. We analyzed DNA damage when PCa cells were treated with SAHA and veliparib alone or in combination, and further explored the molecular mechanisms by which veliparib and SAHA co-target the UHRF1/BRCA1 complex to impair HR DNA damage repair. Eventually, the anticancer efficacy of drug combination was validated in xenograft models.

## Methods

### Reagents

PARP inhibitor veliparib (ABT-88) and HDAC inhibitor SAHA were purchased from Selleck, China (Shanghai, China). Both were dissolved in dimethylsulfoxide (DMSO). Stock solutions were 50 mM for SAHA and 100 mM for veliparib.

### Cell culture

Human PCa cells LNCaP, VCaP, PC-3 and DU145 and non-malignant prostate epithelial cells were purchased from ATCC (Manassas, VA, USA). C4–2 and CWR22Rv1 were obtained from Dr. Chinghai Kao at the Indiana University School of Medicine. PCa cells were maintained in RPMI-1640 medium (Thermo Fisher Scientific, Waltham, MA, USA) supplemented with 10% FBS (Gibco, Thermo Fisher Scientific, Friendship, ME, USA) and penicillin/streptomycin antibiotics. RWPE-1 cells were maintained in defined Keratinocyte-SFM (1×) liquid (Invitrogen, Carlsbad, CA, USA). All cells were cultured with 5% CO2 in a 37 °C incubator.

### Cell viability assay

Cell viability was assessed by crystal violet assay. Cells were plated in 24-well plates with a cell density of 4 × 10^4^ cells per well, and incubated at 37°Cfor 24 h. Then the cells were treated with SAHA and veliparib alone or in combination. Three days after drug treatment, cells were fixed with 4% paraformaldehyde for 30 min and then stained with 0.1% crystal violet solution for 20 min (300 uL/well). After thorough washing with tap water, plates were air-dried for at least 2 h at room temperature. Cells were lysed by shaking the cells in 1% SDS (400uL/well) for 30 min. Cell viability was evaluated by measuring the absorbance of each well at 570 nm with a VersaMax™ microplate reader.

### Clonogenic formation assay

The cells were plated in 6-well plates (500 cells/well) at 37 °C overnight and then treated with SAHA and veliparib alone or in combination. Cell culture media were replaced with fresh growth medium with drugs every 3 days. The cells were fixed and stained with 0.1% crystal violet solution 7 days after drug treatment. Clones with > 50 cells were counted under the microscope and the survival fractions were calculated as the average number of colonies± SD of three independent experiments.

### Apoptosis assays

PCa cells were treated with SAHA and veliparib alone or in combination at the indicated concentrations. Cells were harvested 5 days after drug treatment, EDTA free trypsin and centrifugation at 2000 rpm for 5 min. After washing cells with PBS twice, cell apoptosis was analyzed by two methods: staining the cells with FITC-Annexin V/propidium iodide (PI) at room temperature avoiding light for 10 min, and assessing the sub-G1 cell sub-population after PI staining with flow cytometry. The positive cells were detected by flow cytometer.

### Immunofluorescence

LNCaP cells were maintained in 24-well plates with coverslips and treated with SAHA and veliparib alone or in combination for 48 h. Cells were fixed with 4% paraformaldehyde for 10 min, and permeabilized with 0.1% Triton X-100 for 10 min. The cells then were incubated with γH2AX antibody (phosphoS139, CST, Danvers, MA, USA) at 4 °C overnight. Cells were washed with cold PBS for three times, and then incubated with the second antibody. The nuclei were stained with DAPI. Images were captured and analyzed with Leica fluorescence microscopy.

### Western blot analysis

Protein levels were assessed by western blot. Antibodies against HDAC, PARP, cleaved-PARP, H3, Acetyl-H3, and RAD51 were purchased from Cell Signaling Technology (CST). Anti-UHRF1, Ku-70, ERCC1, MSH2, MSH6, GAPDH and anti-α-tublin antibodies were purchased from Santa Cruz Biotechnology (Dallas, TX, USA). Anti-BRCA1 and phosphorylated-BRCA1(ser988) was purchased from ABclonal Technology (Upper Heyford, UK). Anti-PAR antibody was purchased from Trevigen (4335-MC-100, MD, USA).

### Small RNA interference

Two specific siRNAs targeting 2 UHRF1 sequences were purchased from GenePharma(Shanghai, China). siRNAs were transfected into PCa cells using Dharmafect Transfection Reagents (Lafayette, CO, USA).

### In vivo animal study

The animal experiment protocol had been approved by the Ethics Committee of Xiangya Hospital, Central South University. Twenty nude male mice (5 to 6 week-old) were purchased from the SLAC Laboratory, Shanghai, China. The tumor xenografts were induced by subcutaneously inoculating DU145 cells (5 × 10^6^/100 uL) into the left flank region. Three weeks later, the nude mice bearing tumor xenografts were randomly divided into 4 groups, and received the following treatments for 3 continuous weeks: vehicle, SAHA (25 mg/kg.d,i.p.), veliparib(25 mg/kg.d,oralgavage), SAHA(25 mg/kg.d,i.p) plus veliparib(25 mg/kg.d,oral gavage). Tumor size and body weight were measured every 4 days, and tumor xenograft volume(*V*)was calculated using the following formula: *V* = *ab*^2^/2 (*a*: the long diameter and *b*: the short diameter). The tumor xenografs were isolated at the endpoint of experiment, and the tumor size and weight was compared by using the statistical analysis.

### Statistics

All data were analyzed by Statistical Product and Service Solutions 17.0. Results were presented as Mean ± SD (Standard Deviation) or SEM (Standard error of the mean). One-way ANOVA was used to analyze the statistical difference of multiple groups. *P < 0.05, **P < 0.01 and ***P < 0.001. P < 0.05 was considered as significant.

## Results

### PCa cell sensitivity to SAHA or veliparib-induced cell killing differs

**Fig. 1:**
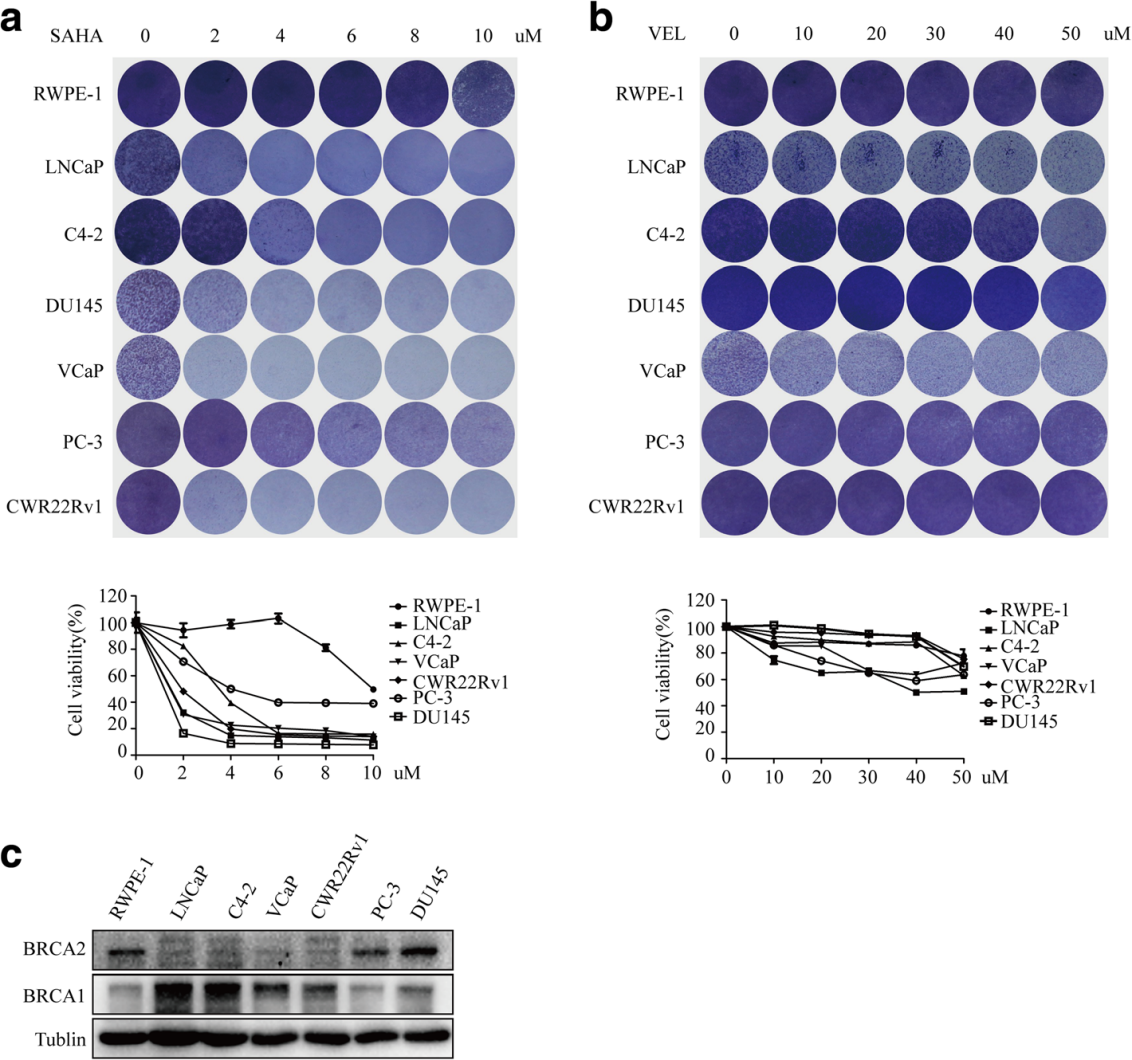
Sensitivity of prostate cancer cells or non-malignant prostate epithelial cells to SAHA or veliparib-induced cell killing. LNCaP, C4–2, DU145, VCaP, PC-3 and CWR22Rv1 PCa cells and non-malignant RWPE-1 prostate epithelial cells were treated with SAHA (**a**) or veliparib (**b**) at increasing concentrations as shown. Cells were stained with crystal violet 3 days after treatment and then lysed with 1% SDS. Cell viability was measured by spectrometer as OD570 nm. Graph represents the mean cell viability ± SD of 3 independent experiments. **c** The protein levels of BRCA1 and BRCA2 were assessed by western blot in PCa cells and non-malignant RWPE-1 prostate epithelial cells

To evaluate the sensitivity of PCa cells to SAHA or veliparib, we treated PCa cells and non-malignant RWPE-1 prostatic epithelial cells with increasing concentrations of SAHA or veliparib. Cell viability was analyzed by crystal violet assay 3 days after treatment. Higher SAHA toxicity was observed in PCa cells than in RWPE-1 cells (Fig. 1a), suggesting high in vitro cancer selectivity of SAHA. By comparison, veliparib did not show noticeable toxicity in all tested cells at concentrations under 50 uM. DU145, a BRCA1 mutation CRPC cell line (Additional file 1: Table S1) showed a higher sensitivity to veliparib. Little difference in veliparib sensitivity was detected between PCa cells and RWPE-1 cells (Fig. 1b). The results indicate that veliparib has low potential to treat PCa unless its cell kill and cancer selectivity can be improved. To learn about the statuses of BRCA1 and BRCA2 in prostate cancer and non-cancerous cells, we assessed their protein levels. BRCA1 and BRCA2 did not exhibit a consistent expression levels in the tested cells (Fig. 1c). Additionally, we collected the mutation profiling of genes from the available data resources, which have been confirmed to induce “BRCAness”. The mutation of BRCA1 had been previously reported in DU145 cells, while the mutation of BRCA2 had been reported in CWR22Rv1 and DU145 cells (Additional file 1: Table S1).

### Co-administration of SAHA and veliparib synergistically improved cell killing and cancer selection in PCa cells

**Fig. 2:**
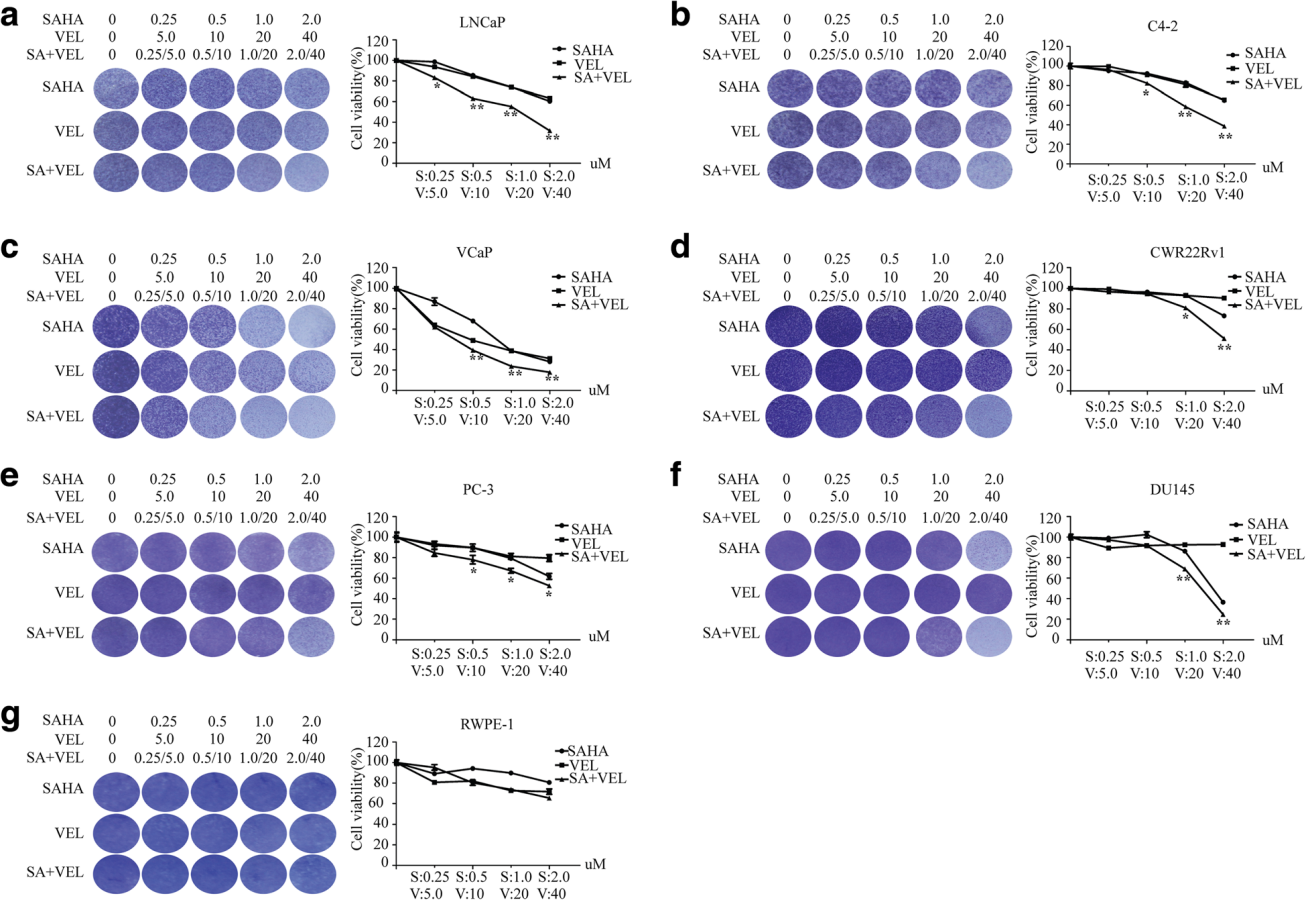
Co-administration of SAHA and veliparib synergistically increased PCa killing. PCa and non-malignant prostate epithelial cells were treated with increased doses of SAHA or veliparib alone or in combination at a constant ratio of 1:20 for 3 days. Representative crystal violet-stained cell images are shown (left). The cells were lysed with 1% SDS, and cell viability was measured by spectrometer as OD570 nm. Graph represents the mean cell viability ± SD of 3 independent experiments.* *p* < 0.05;**p < 0.01(co-treatment vs. SAHA or veliparib alone). **a** LNCaP. **b** C4–2. **c** VCaP. **d** CWR22Rv1. **e** PC-3. **f** DU145. **g** RWPE-1

Due to low toxicity and cancer selection of veliparib in PCa cells at concentrations under 50 uM, we tested whether SAHA significantly promoted veliparib toxicity and cancer selection. To test this hypothesis, we treated PCa cells and RWPE-1 cells with SAHA and veliparib alone or in combination at a constant ratio of 1:20 [31], and assessed cell viability 3 days after drug treatment. SAHA or veliparib alone decreased cell viability, and co-treatment with SAHA and veliparib synergistically improved PCa cell killing (Fig. 2a-f). Non-malignant RWPE-1 prostate epithelial cells showed no obvious toxicity from SAHA or veliparib, singly or in combination (Fig. 2g).

To test the synergistic cell killing effect of SAHA and veliparib co-administration, we calculated the combination index (CI) value for an effective dose for 50% (IC50) in PCa cells and RWPE-1 cells. Co-treatment with SAHA and veliparib synergistically promoted PCa cell killing (CI < 1), but not RWPE-1 cells (CI = 1.05). The HDAC inhibitor SAHA significantly promoted veliparib cell killing and cancer selectivity (Table 1).

**Table 1:** Co-effect of SAHA and veliparib

Cell lines (SAHA: Veliparib)	CI values (IC_50_)
LNCap (1:20)	0.86
C4–2 (1:20)	0.95
VCaP (1:20)	0.90
CWR22Rv1 (1:20)	0.85
PC-3 (1:20)	0.74
DU145 (1:20)	0.85
RWPE-1 (1:20)	1.05

IC_50_: Half maximal inhibitory concentration

CI value: Combination index value

CI < 1 indicates synergy; CI = 1 indicates addictivity; CI > indicates antagonism

### Co-administration of SAHA and veliparib synergistically decreased PCa cell colony formation efficiency

**Fig. 3:**
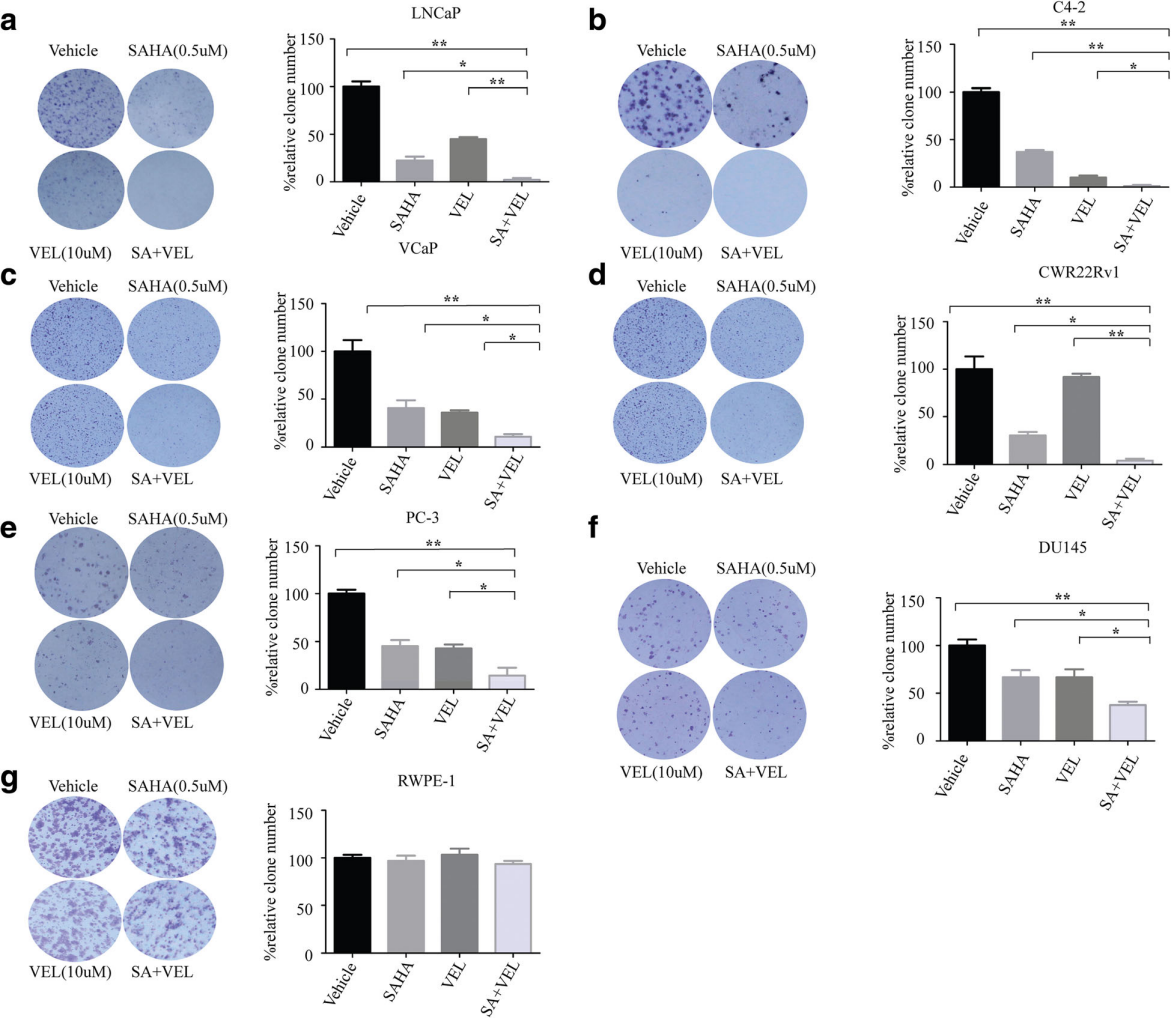
Co-administration of SAHA and veliparib synergistically decreased PCa colony formation. Prostate cancer and non-malignant prostate epithelial cells were treated with SAHA (0.5 uM) and veliparib (10 uM) alone or in combination for 7 days. The cell colonies were stained by crystal violet assay. A representative image is shown. The number of cell colonies was counted. Mean relative percent of clone number ± SD is shown.*p < 0.05;**p < 0.01 (SAHA or veliparib alone vs. co-treatment). **a** LNCAP. **b** C4–2. **c** VCaP. **d** CWR22Rv1. **e** PC-3. **f** DU145. **g** RWPE-1

Since co-treatment with SAHA and veliparib showed synergistic cell killing effects, we validated the synergy of SAHA and veliparib in a clonogenic survival assay. PCa cells were treated with SAHA and veliparib alone or in combination with cells plated at the same numbers. The number of cell colonies represented the sensitivity of PCa cells. Compared to the control, SAHA or veliparib remarkably reduced the number of cell colonies, and co-treatment with SAHA and veliparib further reduced clonogenicity compared to the single drugs (Fig. 3a-f). No significant difference in clonogenicity was found for RWPE-1 cells between the co-treatment and single drug administration groups (Fig. 3g).

### Co-administration of SAHA and veliparib selectively induced PCa cell apoptosis

**Fig. 4:**
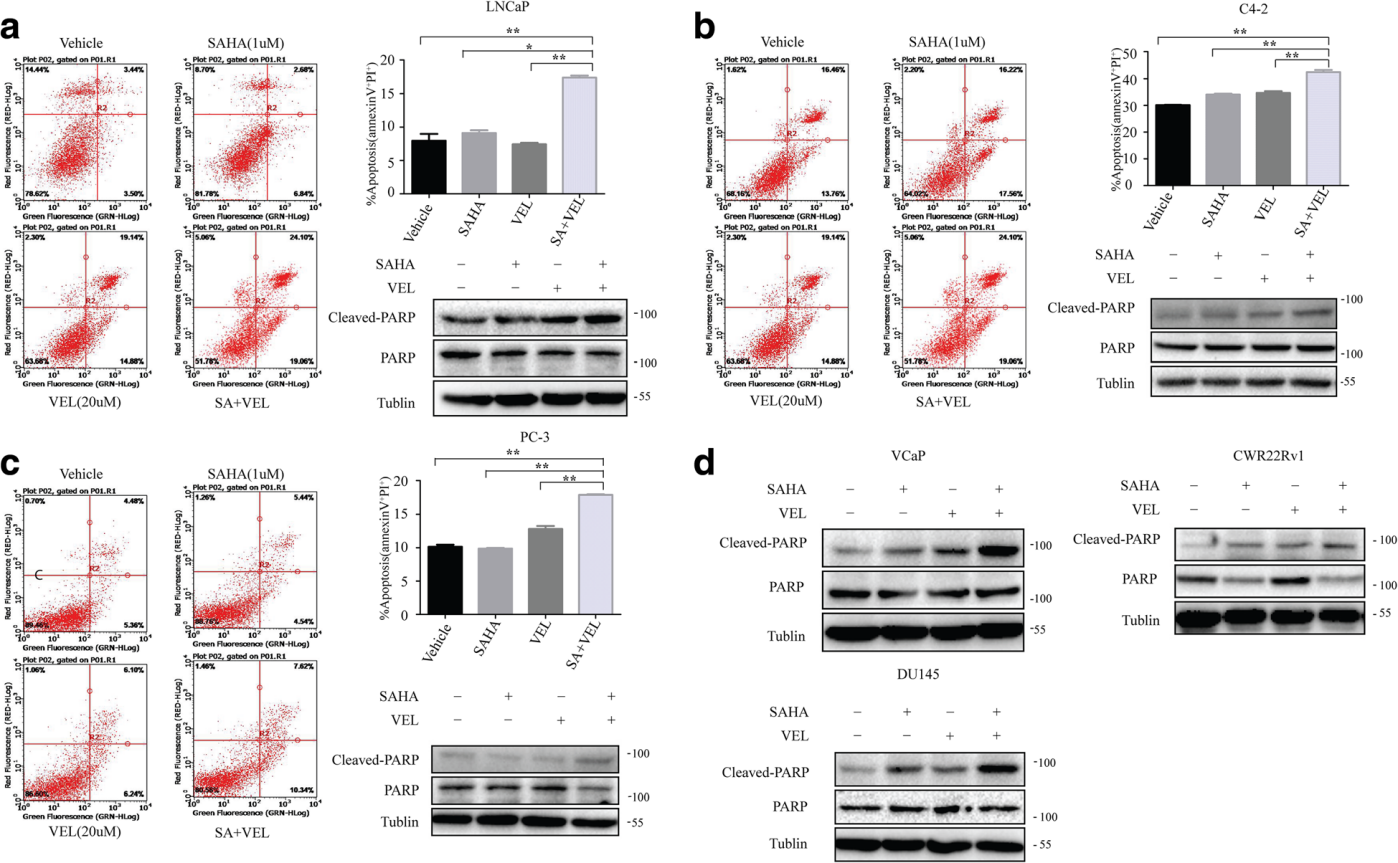
Co-administration of SAHA and veliparib enhanced PCa cell apoptosis. PCa cells LNCaP, C4–2 and PC-3 were treated with SAHA and veliparib alone or in combination at the indicated doses for 4 days (**a** LNCaP. **b** C4-2. **c** PC-3. **d** VCap, CWR22Rv1, DU145). Cells were stained with FITC-Annexin V antibody and counterstained with PI. The apoptotic cells were analyzed by flow cytometery. Representative dot plots of FITC-Annexin V/PI staining are shown. Graph shows mean apoptotic cells (Annexin-V^+^/PI^+^) ± SD. Experiments were performed in triplicate. Cell apoptosis was validated by testing the protein levels of cleaved PARP by western blotting (**a**-**d**). *p < 0.05; ** *p* < 0.01 (SAHA or Veliparib alone vs. co-treatment)

To investigate whether SAHA and veliparib caused synergistic cell killing by inducing more cell apoptosis, we labeled cells with FITC Annexin V/PI, and assessed cell apoptosis by flow cytometry when PCa cells were treated with SAHA (1uM) and veliparib (20uM) alone or in combination (Fig. 4a-c and Additional file 2: Figure S1A-B). The results showed that a combination of SAHA and veliparib induced more cell apoptosis than SAHA or veliparib alone in LNCaP, C4–2 and PC-3 cells. To further confirm the results, we tested the level of cleaved PARP, which represents cell apoptosis, in all tested PCa cells. Results showed that co-treatment with SAHA and veliparib significantly increased the levels of cleaved PARP compared to SAHA or veliparib alone (Fig. 4a-d). These findings showed that SAHA augmented the cell killing activity of veliparib in PCa cells by inducing the increase of cell apoptosis.

### SAHA and veliparib synergistically induced DNA damage of PCa cells

**Fig. 5:**
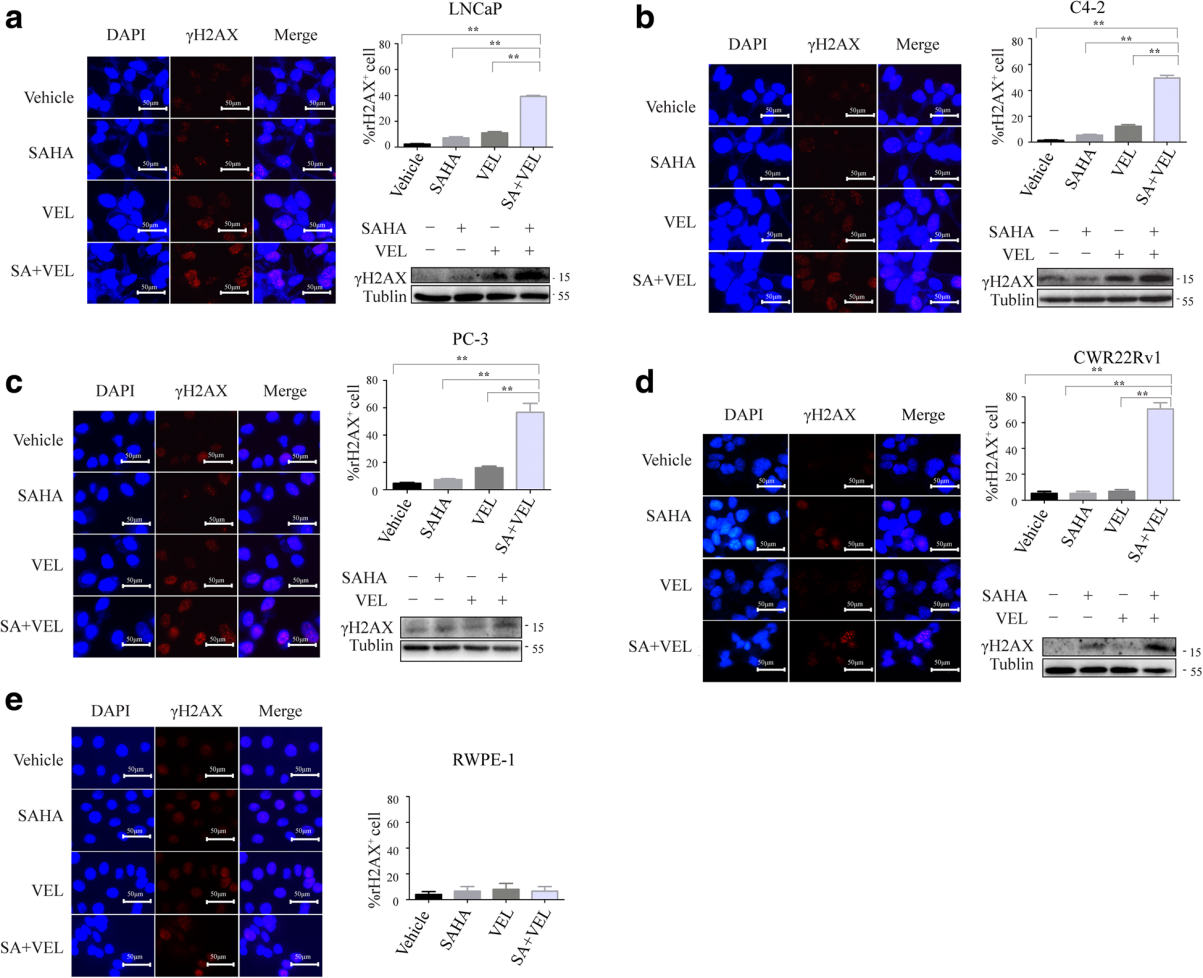
Co-administration of SAHA and veliparib increased DNA damage in PCa cells. PCa cells and non-malignant prostate epithelial cells were treated with SAHA (1uM) and veliparib (20uM) alone or in combination, and cells were stained with immunofluorescent γH2AX antibody to show DNA damage foci (red punctate staining) at the site of DSBs. Nuclei were counterstained with DAPI. Percentage of γH2AX^+^ cells ± SD is shown.*p < 0.05;**p < 0.01 (SAHA or veliparib alone vs. co-treatment). Immunofluorescence was validated by testing the increased γH2AX protein expression. **a** LNCaP. **b** C4–2. **c** PC-3. **d** CWR22Rv1. **e** RWPE-1

Veliparib, as a PARP inhibitor, exerts its anticancer effect by inducing DNA damage. Since HDAC inhibitor SAHA has been reported to enhance olaparib cell kill by inducing even more severe DNA damage [31], it is possible that SAHA enhanced veliparib cell kill in similar pathways. Formation of γH2AX foci is a well-known marker of DNA damage. When double-strand DNA breaks (DSBs) occur, H2AX protein is phosphorylated at residue serine 139(c), and γH2AX forms foci at chromosomal sites of DSBs [38], which can be detected by fluorescence microscopy. SAHA or veliparib induced γH2AX foci formation, as shown by the punctate staining in the nuclei. SAHA and veliparib co-treatment induced many more γH2AX foci. The results indicated that co-treatment with SAHA and veliparib induced more severe DNA damage than SAHA or veliparib alone (Fig. 5a-d). To test whether SAHA and veliparib selectively induced DNA damage in PCa cells but not normal prostate epithelial cells, we treated RWPE-1 cells with SAHA and veliparib alone or in combination. Many fewer punctuate γH2AX foci were detected in the nuclei of RWPE-1 cells (Fig. 5e). Consistently, the combination of SAHA and veliparib significantly increased γH2AX protein levels. These results showed that the co-treatment with SAHA and veliparib significantly promoted DNA damage compared to SAHA or veliparib alone.

### SAHA and veliparib synergistically decreased the expression of UHRF1 and HR DNA repair protein BRCA1

**Fig. 6:**
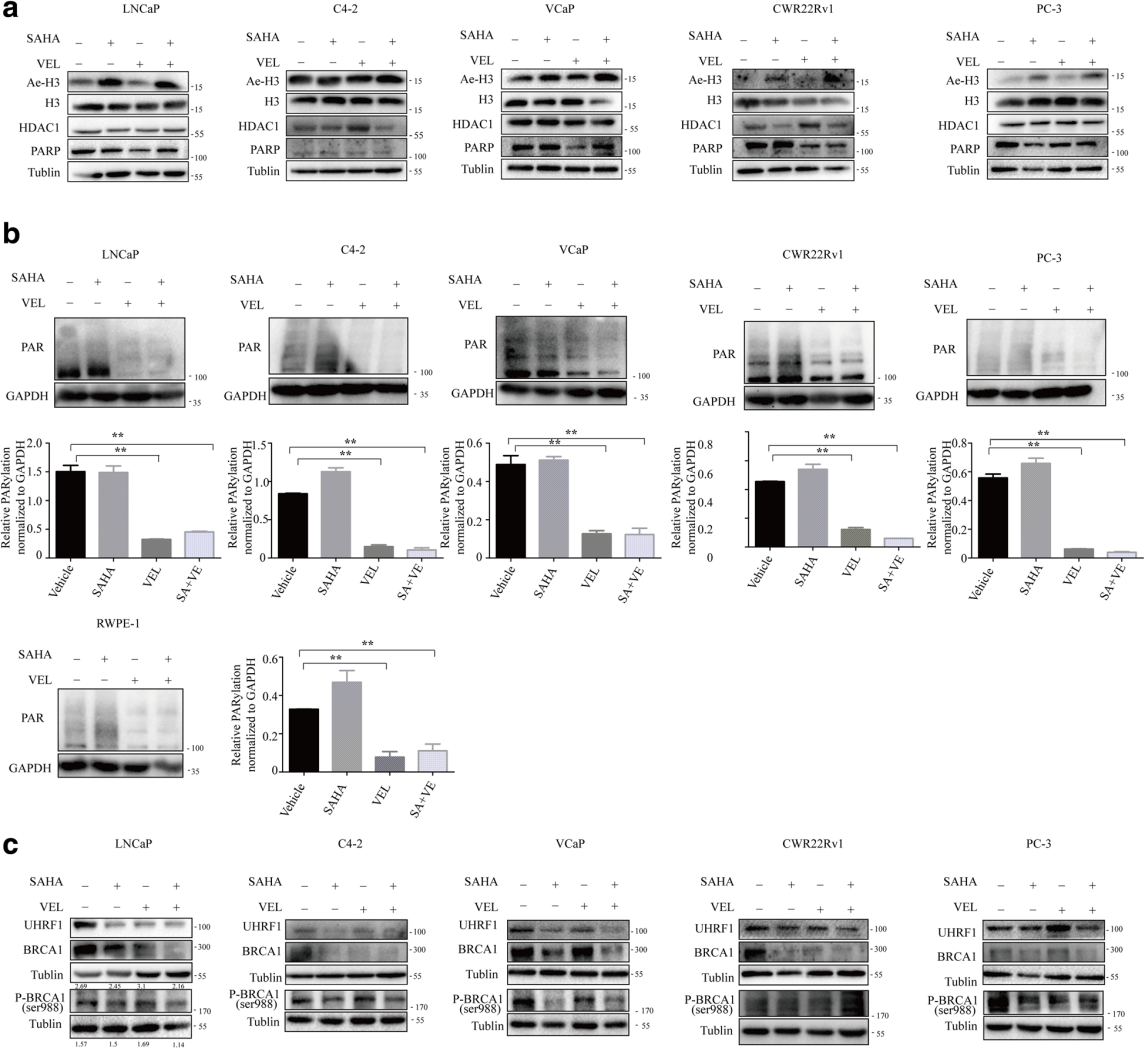
Co-treatment of SAHA and veliparib decreasedtheprotein levels ofUHRF1 and BRCA1. LNCaP, C4–2, VCaP, CWR22Rv1 and PC-3 PCa cells were treated with SAHA (1uM) and veliparib (20uM) alone or in combination. **a** Protein levels of acetylated histone 3, histone 3, PARP and HDAC1 were tested when the PCa cells were treated with SAHA and veliparib singly or in combination. **b** The levels of PAR was determined by western blot after drug treatment. Veliparib shows significant anti-PARP activity in PCa cells and non-malignant prostate epithelial cell. **c** Protein levels of UHRF1, BRCA1 and phosphorylated BRCA1(ser988) were assessed by western blot 3 days after drug treatment

SAHA, a HDAC1 inhibitor, significantly increased the protein level of acetylated H3 protein in all tested PCa cells, showing the inhibition efficiency on HDAC, but no effect on the levels of PARP and HDAC1 (Fig. 6a). Veliparib, as a PARP inhibitor, shows significant anti-PARP activity in PCa cells and prostate normal epithelial cells (Fig. 6b). These data validated the therapeutic effect of SAHA and veliparib on PCa cells. It was previously reported that SAHA or olaparib reduced the levels of such HR DNA damage repair proteins as RAD51 and BRCA1, and co-treatment with both drugs synergistically reduced RAD51 protein, but showed no synergistic reduction for BRCA1 protein [4]. In the present study we observed alternative effects when the cells were treated with SAHA and veliparib. The administration of single drug reduced the protein levels of RAD51 to different extents, but co-treatment did not show synergistic effect (Additional file 3: Figure S2A). In addition to HR DNA damage repair proteins, we tested other DNA damage repair pathways, such as ERCC1 for nucleotide excision repair (NER), KU70 (XRCC6) for Non-homologous end joining (NHEJ), MSH2 and MSH6 for mismatch repair (MMR). The single or combined treatment of SAHA and veliparib did not consistently change these tested DNA repair pathways (Additional file 3: Figure S2B). Meanwhile, consistently in five tested PCa cell lines, co-treatment with SAHA and veliparib synergistically reduced UHRF1 and BRCA1 proteins, but shows no effect on the phosphorylated BRCA1 (Fig. 6c). These data suggest that the DNA damage repair pathways affected by the combination of veliparib with SAHA are different from those affected by SAHA and olaparib. The data suggest that the synergistic effect of two drugs on BRCA1 is only a contributing factor for DNA damage repair, and PARP inhibitor per se certainly influence DNA damage repair pathways in individual manners.

### SAHA and veliparib synergistically destroyed the protein stability of UHRF1 and BRCA1

**Fig. 7:**
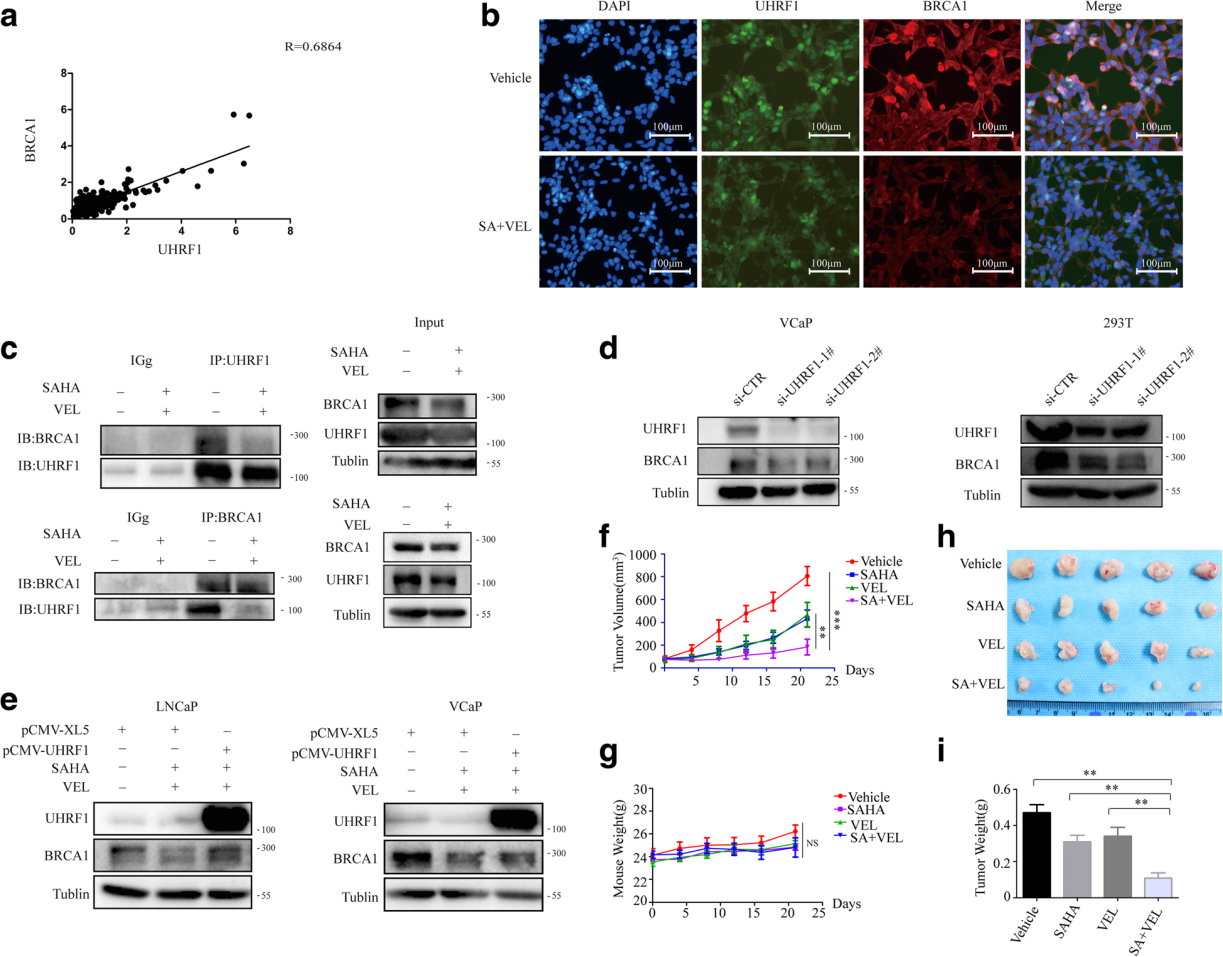
SAHA and veliparibsynergistically destroyed the protein stability of UHRF1 and BRCA1. **a** The mRNA expression of UHRF1 and BRCA1 showed a high positive correlation in 495 PCa samples from TCGA data (*R* = 0.6864). **b** Co-treatment with SAHA and veliparib decreased co-localization of UHRF1 and BRCA1 proteins. LNCaP cells were treated with SAHA (1uM) and veliparib (20uM) for 48 h, and then reacted with UHRF1 and green fluorescent secondary antibodies or BRCA1 and red fluorescent secondary antibodies. The nuclei were labeled with DAPI. The co-localization of UHRF1 and BRCA1 was observed with a fluorescence microscopy. **c** Co-treatment with SAHA and veliparib decreased the protein interaction of BRCA1 and UHRF1. UHRF1 and BRCA1 were up-regulated in HEK-293 cells by transient transfection. The HEK-293 and Hela cells were treated with or without SAHA and veliparib, UHRF1 protein was co-immunoprecipitated (co-IP), and BRCA1 protein was identified in the protein complex by western blot. **d** BRCA1 protein levels were tested when UHRF1 was depleted with siRNA in VCaP and HEK-293 cells. **e** BRCA1 protein levels were tested when LNCaP and VCaP cells were co-treated with SAHA and veliparib with or without the elevation of UHRF1 level. **f** DU145 tumor xenografts were induced in nude mice, and then were treated with vehicle or drugs for continuous 3 weeks. The volumes of xenografts were monitored (**P < 0.01,*P < 0.05, compared to other three group). **g** The body weight of nude mice was monitored during the entire treatment. **h** and **i** The tumors were isolated at the endpoint of experiment, and the size and weight of tumors were compared

It is fascinating that SAHA and veliparib synergistically reduced UHRF1 protein levels consistently with BRCA1. We analyzed the mRNA expression data of 495 PCa samples from the Cancer Genome Atlas (TCGA) database. The mRNA levels of BRCA1 and UHRF1 demonstrated a high positive correlation (*R* = 0.6864, Fig. 7a). We observed the co-localization of UHRF1 and BRCA1 proteins in LNCaP cells using cell immunofluorescence (Fig. 7b). It has been reported that BRCA1 recruits UHRF1 protein to the sites of DSBs in S phase through protein interaction [37].We suggest that co-treatment with SAHA and veliparib synergistically inhibited DNA damage repair by destroying the protein interaction of UHRF1 and BRCA1. Co-treatment with SAHA and veliparib synergistically reduced the protein co-localization of UHRF1 and BRCA1 by cell immunofluorescence (Fig. 7b). The results were further validated by co-immunoprecipitation in exogenous HEK293 cells with UHRF1 and BRCA1 overexpression, and in endogenous HeLa cells (Fig. 7c).

We further tested whether co-treatment reduced BRCA1 protein through UHRF1. We assessed the protein levels of BRCA1 when UHRF1 was depleted with siRNAs. UHRF1 depletion significantly reduced BRCA1 protein levels (Fig. 7d), but BRCA1 depletion did not reduce UHRF1 protein (data not shown). We further elevated UHRF1 levels in LNCaP or VCaP cells by transient transfection, followed by treatment with SAHA and veliparib. The reduction in BRCA1 protein levels was rescued by UHRF1 elevation (Fig. 7e). These data showed that SAHA and veliparib synergistically inhibited DNA damage repair by destroying the protein stability of BRCA1 through UHRF1, thereby promoting DNA damage and cell death.

We validated the in vivo synergistic efficacy of the co-treatment of SAHA and veliparib in the tumor xenograft models derived from DU145 cells. Consistent to the in vitro data, SAHA or veliparib alone inhibited tumor growth, and the co-treatment synergistically inhibited tumor growth (Fig. 7f, h and i). Furthermore, the drug treatments did not make detectable toxicity on nude mice, as shown the body weight shad no difference after treatment (Fig. 7g).

## Discussion

Veliparib is a PARP inhibitor now being tested for safety and anticancer efficacy in a number of clinical trials, including PCa trials. Although PARP inhibitors have shown great success for BRCA-mutated tumors, patients still develop acquired drug resistance. Several possible mechanisms have been proposed. Drug efflux through transporters decreases drug intake [39]. The decline or loss of PARP1 expression decreases drug response. PARP1 has been reported the involvement in several DNA repair pathways [40], including base excision repair (BER) [41] or single-strand break repair (SSBR) by binding to apurinic/apyrimidinic (AP) sites [42] or recruiting BER complex [43], promotes nucleotide excision repair (NER) or mismatched repair by ADP-ribosylates XPA [44] or MSH6 [45], and promotes NHEJ by activating DNA-PKcs [44, 45]. In addition, PARP1 promotes homologous recombination (HR) by activating ATM signaling [46]. Cancer patients develop secondary genetic or epigenetic mutations restoring functional HR in tumors that were formerly HR deficient [47, 48], or restore BRCA protein function [49] after treatment. BRCA1/2 is involved in homologous recombination (HR) DNA damage repair by forming different protein complexes with CCDC98, RBBP8 and BACH1 at different stages of double strand DNA break repair (DBS) [50, 51]. In BRCA-mutated cancer cells, PARP inhibitors further inhibit DNA damage repair, leading to cell death. In addition, somatic mutations of TP53BP1 induce partial restoration of HR [52].

Improving the cell killing and cancer selectivity of veliparib apparently requires a novel drug combination. Recent studies showed that PCa with HR deficiency may be sensitive to PARP inhibitors and platinum chemotherapy [53, 54]. In this study, we observed that SAHA or veliparib alone decreased cell viability and clonogenicity, and induced cell apoptosis and DNA damage. Importantly, co-administration of the two drugs synergistically decreased cell viability and clonogenicity and enhanced cell apoptosis and DNA damage, with no detectable toxicity to normal prostate epithelial cells at the tested dose window.

It has been reported that the PARP inhibition down-regulates BRCA1 in a pathway mediated by E2F4 and p130 [55]. Other publication suggested that HDAC inhibition down-regulates BRCA1 mRNA level by decreasing the amount and recruitment of E2F1 transcription factor [27]. In our present manuscript, we proposed a novel mechanism by which two drugs further induced BRCA1 protein degradation. UHRF1 is an important epigenetic regulator that has been implicated in treatment resistance [56–58] and DNA damage repair [59]. It is a binding factor for DNA interstrand crosslik lesions (ICL), and is involved in processing ICL lesions by recruiting structure-specific endonucleases [36]. PARP1 interacts with UHRF1 protein [60], suggesting that UHRF1 is involved in single strand DNA damage repair. UHRF1 plays a double-facet role in the regulation of BRCA1, i.e. UHRF1 silences BRCA1 gene transcription, and sustains BRCA1 protein stability. On one hand, UHRF1 as an epigenetic regulator, together with other enzymes including histone deacetylase 1 (HDAC1), DNA methyltransferase 1 (DNMT1) and histone lysine methyltransferases G9a and Suv39H1 caused the epigenetic silencing of tumor suppressor genes including BRCA1 by inducing DNA methylation and histone post-translation modification changes [61]. On the other hand, when DSB damage occurs, BRCA1 recruits UHRF1 to the damage site and mediates RIF degradation to help DNA repair [37], and UHRF1 is required for the maintenance of protein stability of BRCA1. Deficiency of UHRF1 function promotes DNA damage sensitivity [37]. Targeting UHRF1 may be an attractive strategy to improve the anticancer efficiency of PARP inhibitors. However, an effective UHRF1 inhibitor is still unavailable for pre-clinical and clinical studies [34].

UHRF1 protein interacts with HDAC proteins in the epigenetic repression complex [62]. The pan-HDAC inhibitor SAHA induced the acetylation of histone protein H3 (Fig. 6a), and induced the degradation of UHRF1 protein. We report for the first time that SAHA or veliparib alone decreased UHRF1 and BRCA1 protein levels to different extents, and that co-treatment with SAHA and veliparib consistently and synergistically decreased UHRF1 and BRCA1 protein levels. Since UHRF1 protein physically interacts with BRCA1, the depletion of UHRF1 decreased BRCA1 protein levels. Thus co-treatment with SAHA and veliparib decreased the protein levels of BRCA1 through UHRF1 (Fig. 7d). However, co-treatment did not reduce RAD51 protein levels. The synergistic reduction of BRCA1 protein in response to the combination of veliparib and SAHA is different from the previous report in which olaparib and SAHA induced a synergistic reduction of RAD51 but not BRCA1 [31]. The mechanism for this difference is worth further study, though the PARP inhibitor per se certainly influences the pathways of DNA damage repair.

## Conclusion

**Fig. 8:**
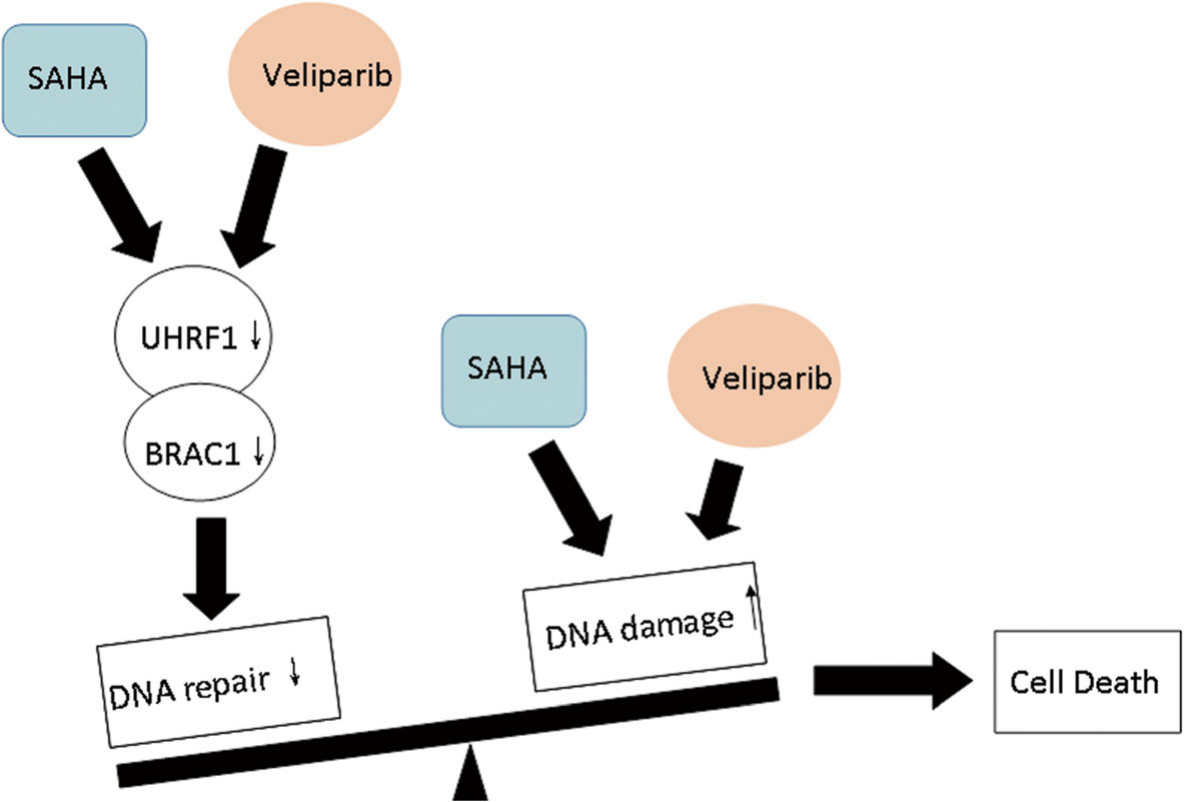
Working model of SAHA and Veliparib co-treatment. SAHA and veliparib exhibited synergistic anticancer efficacy in prostate cancer cells by two mechanisms: synergistically inducing DNA damage, and impairing DNA damage repair by reducing HR DNA repair molecule BRCA1 levels via UHRF1. Co-treatment promoted DNA damage and impaired the damaged DNA repair, and enhanced cell apoptosis and cell death

BRCA1 is a key protein for HR DNA damage repair, and the HDAC and PARP inhibitors SAHA and veliparib promoted DNA damage by co-inhibiting BRCA1 via UHRF1, targeting the UHRF1/BRCA1 protein complex (Fig. 8). This combination strategy has noteworthy potential for future clinical trials as a PCa treatment.

## Additional files

Additional file 1: **Table S1.** Genes Confirmed to Induce BRCAness in Prostate Non- and Cancerous Cell Lines. (DOC 51 kb)

Additional file 2: **Figure S1.** Co-treatment with SAHA and veliparib enhanced cell apoptosis in PCa cells. LNCaP (A) and C4–2(B) cells were treated with SAHA and veliparib alone or in combination for 3 days. Cells were stained with PI, and the apoptotic cells (sub-G1 population) were analyzed by flow cytometry. Graphs show the mean percentage of cells in sub-G1. (TIF 2355 kb)

Additional file 3: **Figure S2**. (A) LNCaP, PC-3, CWR22Rv1 and C4–2 cells were treated with SAHA and veliparib alone or in combination for 3 days. The protein levels of RAD51 were assessed by western blot. (B) LNCaP, C4–2, VCaP, CWR22Rv1 and PC-3 cells were treated with SAHA and veliparib alone or in combination for 3 days. The protein levels of DNA damage repair molecules (Ku-70, ERCC1,MSH2 and MSH6) were assessed by western blot. (TIF 2690 kb)

## Abbreviations

ADT: Androgen deprivation therapy
CST: Cell Signaling Technology
DMSO: Dimethylsulfoxide
DSB: DNA double strand break
HDAC: Histone deacetylase
HR: Homologous recombination
ICL: DNA interstrand crosslink lesions
mCRPC: Metastatic castration-resistant prostate cancer
PARP: Poly ADP-ribose polymerase
PCa: Prostate cancer
PTEN: Phosphatase and tensin homolog
SD: Standard Deviation
SEM: Standard error of the mean
UHRF1: Ubiquitin-like with PHD and ring-finger domain 1
