# Correction to: PARP inhibitor veliparib and HDAC inhibitor SAHA synergistically co-target the UHRF1/. BRCA1 DNA damage repair complex in prostate cancer cells

**DOI:** 10.1186/s13046-022-02290-9

**Published:** 2022-02-22

**Authors:** Linglong Yin, Youhong Liu, Yuchong Peng, Yongbo Peng, Xiaohui Yu, Yingxue Gao, Bowen Yuan, Qianling Zhu, Tuoyu Cao, Leye He, Zhicheng Gong, Lunquan Sun, Xuegong Fan, Xiong Li

**Affiliations:** 1 Center for Molecular Medicine, Xiangya Hospital, Central South University, 87 Xiangya Road, Changsha, 410008 Hunan China; 2 Hunan Key Laboratory of Molecular Radiation Oncology, Xiangya Hospital, Central South University, Changsha, China; 3 State Key Laboratory of Chemo/Biosensing and Chemometrics, Hunan University, Changsha, China; 4 Research Institute for Prostate Disease, Central South University, Changsha, China; 5 Department of Pharmacy, Xiangya Hospital, Central South University, Changsha, China; 6 Hunan Key Laboratory of Viral Hepatitis, Xiangya Hospital, Central South University, Changsha, China

**Correction to: J Exp Clin Cancer Res 37, 153 (2018)**

**https://doi.org/10.1186/s13046-018-0810-7**

Following publication of the original article [1], the authors identified a minor error in Fig. 4; specifically:

- Fig. 4 b: Incorrect flow cytometry graphs of VEL (20uM) and SA + VEL were used; the figure has been corrected to use the correct graphs

**Fig. 4:**
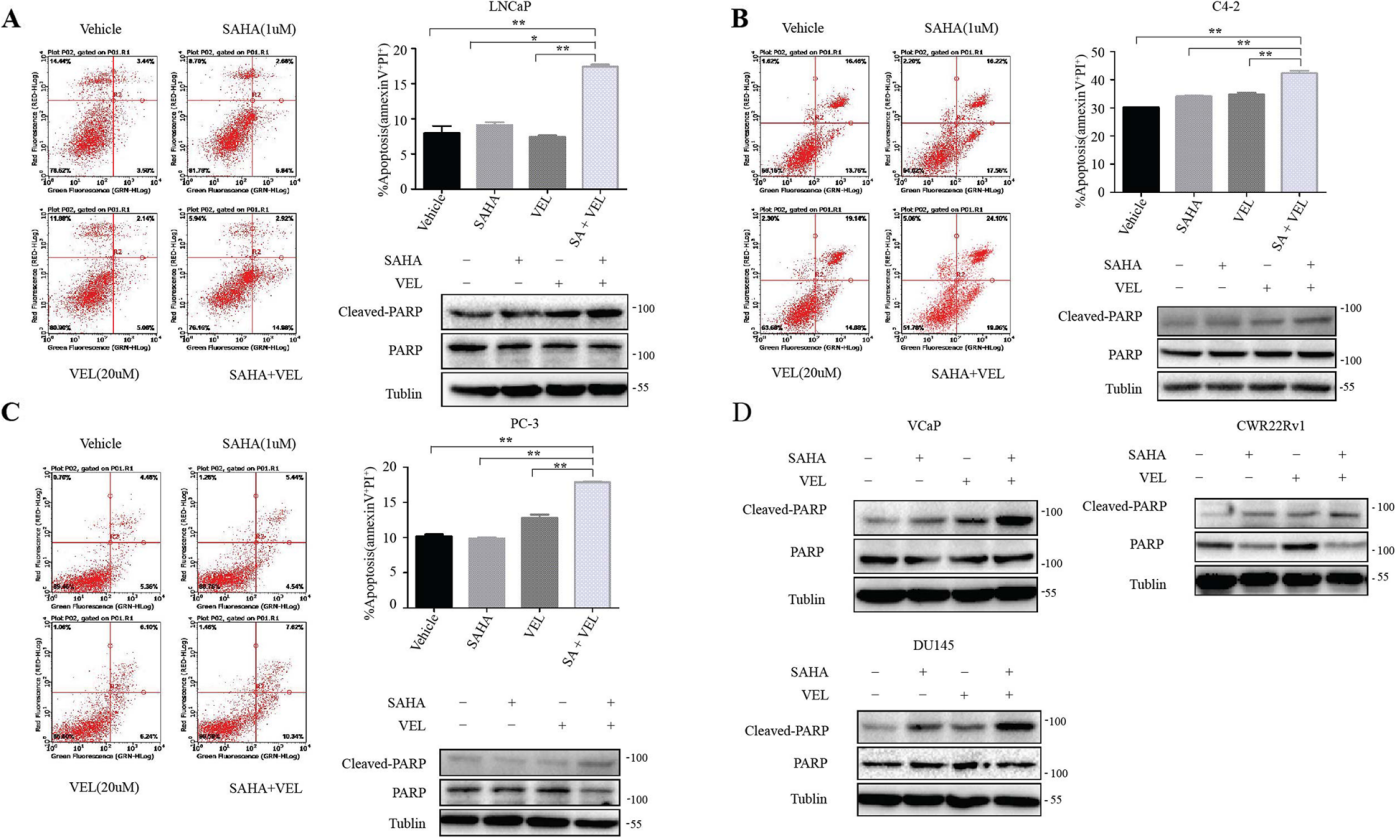
Co-administration of SAHA and veliparib enhanced PCa cell apoptosis. PCa cells LNCaP, C4–2 and PC-3 were treated with SAHA and veliparib alone or in combination at the indicated doses for 4 days (**a** LNCaP. **b** C4–2. **c** PC-3. **d** VCap, CWR22Rv1, DU145). Cells were stained with FITC-Annexin V antibody and counterstained with PI. The apoptotic cells were analyzed by flow cytometery. Representative dot plots of FITC-Annexin V/PI staining are shown. Graph shows mean apoptotic cells (Annexin-V+/PI+) ± SD. Experiments were performed in triplicate. Cell apoptosis was validated by testing the protein levels of cleaved PARP by western blotting (**a-d**). *p < 0.05; ** *p* < 0.01 (SAHA or Veliparib alone vs. co-treatment)

The corrected figure is given here. The correction does not have any effect on the final conclusions of the paper.
