# Supplementary material for: PARP inhibitor veliparib and HDAC inhibitor SAHA synergistically co-target the UHRF1/BRCA1 DNA damage repair complex in prostate cancer cells
Source: J Exp Clin Cancer Res. 2018 Jul 16;37:153. doi: 10.1186/s13046-018-0810-7 (PMC6048811; doi:10.1186/s13046-018-0810-7)
Supplement: Supplementary file 1 — Table S1. Genes Confirmed to Induce BRCAness in Prostate Non- and Cancerous Cell Lines. (DOC 51 kb) [file 13046_2018_810_MOESM1_ESM.doc]

Table 1. Genes Confirmed to Induce BRCAness in Prostate Non- and Cancerous Cell Lines

| Cell lines | Genes inducing BRCAness | Census Gene | AA Mutation | CDS Mutation | Somatic status | Zygosity | Validated | Type | Position |
| --- | --- | --- | --- | --- | --- | --- | --- | --- | --- |
| RWPE1 |  |  |  |  |  |  |  |  |  |
| LNCaP | ATM | Yes | p.Q912* | c.2734C>T | Unknown | Heterozygous | Unverified | Substitution - Nonsense | 11:108139232..108139232 |
|  | ATR | Yes | p.S1493S | c.4479A>T | Unknown | Heterozygous | Unverified | Substitution - coding silent | 3:142234261..142234261 |
|  | ATR | Yes | p.K1379N | c.4479T>A | Unknown | Heterozygous | Unverified | Substitution -  Missense | 3:142242850..142242850 |
|  | BRIP1 | Yes | p.N306N | c.918G>A | Unknown | Heterozygous | Unverified | Substitution - coding silent | 17:59885828..59885828 |
|  | CHEK2 | Yes | p.T387N | c.1160G>T | Unknown | Heterozygous | Verified | Substitution - Missense | 22:29091797..29091797 |
|  | FANCA | Yes | p.E369D | c.1107C>A | Unknown | Heterozygous | Unverified | Substitution - Missense | 16:89858453..89858453 |
|  | RAD51B | Yes | p.S365S | c.52A>T | Previously Reported | Heterozygous | Verified | Substitution - Silent | 14:69077781..69077781 |
|  | PTEN | Yes | p.K6fs*4 | c.16_17delAA | Previously Reported | Heterozygous | Verified | Deletion - Frameshift | 10:89624242..89624243 |
|  | CHD1 | No | p.N310fs*12 | c.922_923insT | Unknown | Heterozygous | Verified | Insertion - Frameshift | 5:98235339..98235340 |
|  | CHD1 | No | p.K401N | c.1203C>G | Unknown | Heterozygous | Unverified | Substitution - Missense | 5:98234122..98234122 |
| VCaP | MSH6 | Yes | p.T1085fs | insC |  |  |  | Insertion - Frameshift | 2:48030639..48030640 |
| CWR22Rv1 | BRCA2 | Yes | p.V1810I | c.5428G>A | Previously Reported | Heterozygous | Unverified | Substitution - Missense | 13:32913920..32913920 |
|  | BRCA2 | Yes | p.T3033fs*11 | c.9097_9098insA | Unknown | Heterozygous | Verified | Insertion - Frameshift | 13:32954022..32954023 |
|  | CHD1 | No | p.E1321fs*22 | c.3960delA | Previously Reported | Heterozygous | Verified | Deletion - Frameshift | 5:98870705..98870705 |
| PC-3 |  |  |  |  |  |  |  |  |  |
| DU145 | BRCA1 | Yes | p.E962K | c.2884C>T | Previously Reported | Heterozygous | Verified | Substitution - Missense | 17:41244664..41244664 |
|  | BRCA2 | Yes | p.S2284L | c.6851C>T | Unknown | Homozygous | Unverified | Substitution - Missense | 13:32918704..32918704 |
|  | BRIP1 | Yes | p.T132N | c.395G>T | Unknown | Heterozygous | Unverified | Substitution - Missense | 17:59926602..59926602 |
|  | CHD1 |  | p.N186fs | insT |  |  |  | Insertion - Frameshift | 5:98236919..98236920 |

Data resources: https://portals.broadinstitute.org/ccle
