# Supplementary figures and images for: PARP inhibitor veliparib and HDAC inhibitor SAHA synergistically co-target the UHRF1/BRCA1 DNA damage repair complex in prostate cancer cells
Source: J Exp Clin Cancer Res. 2018 Jul 16;37:153. doi: 10.1186/s13046-018-0810-7 (PMC6048811; doi:10.1186/s13046-018-0810-7)

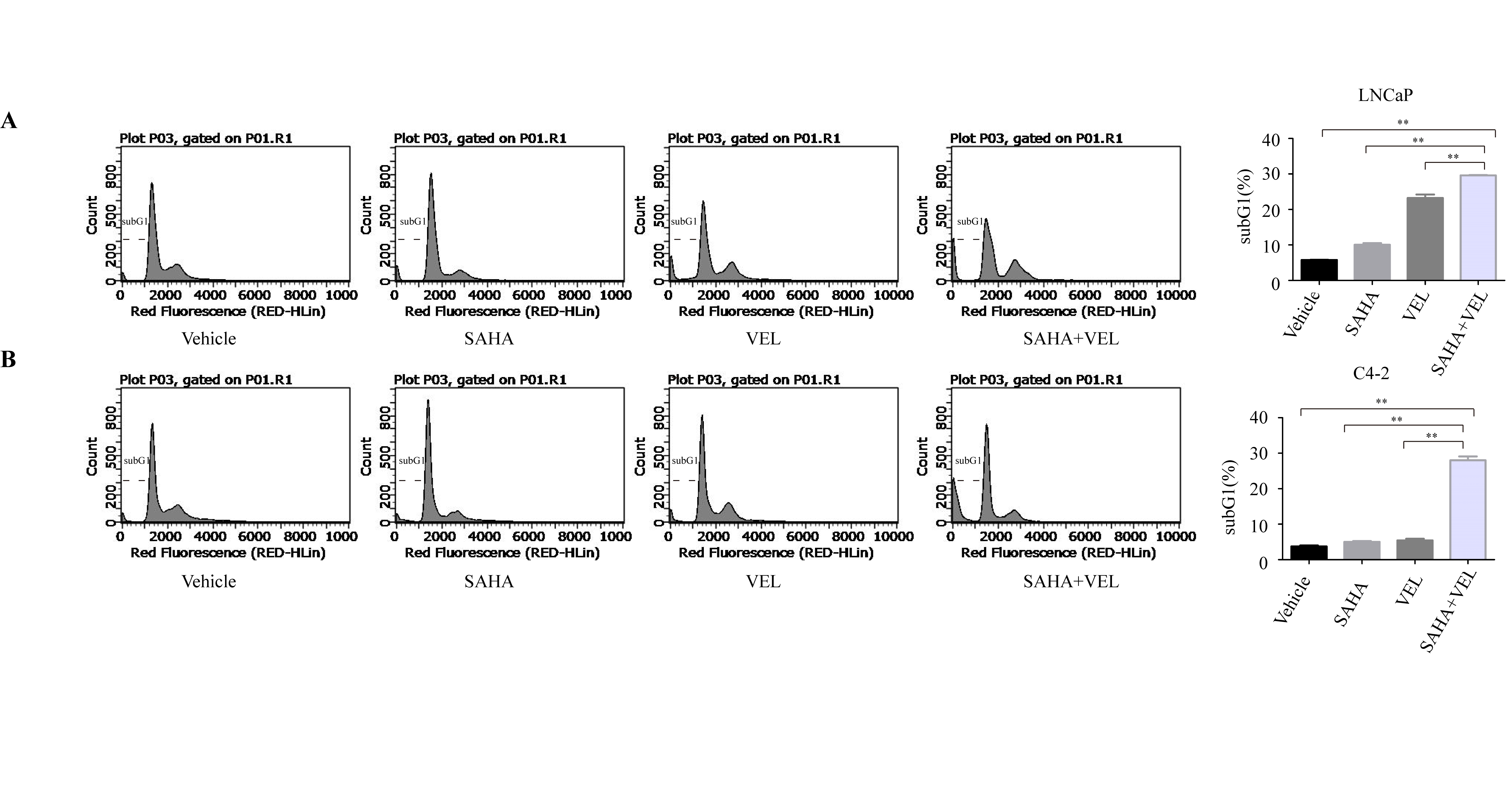

Supplement: Supplementary file 2 — Figure S1. Co-treatment with SAHA and veliparib enhanced cell apoptosis in PCa cells. LNCaP (A) and C4–2(B) cells were treated with SAHA and veliparib alone or in combination for 3 days. Cells were stained with PI, and the apoptotic cells (sub-G1 population) were analyzed by flow cytometry. Graphs show the mean percentage of cells in sub-G1. (TIF 2355 kb) [file 13046_2018_810_MOESM2_ESM.tif]

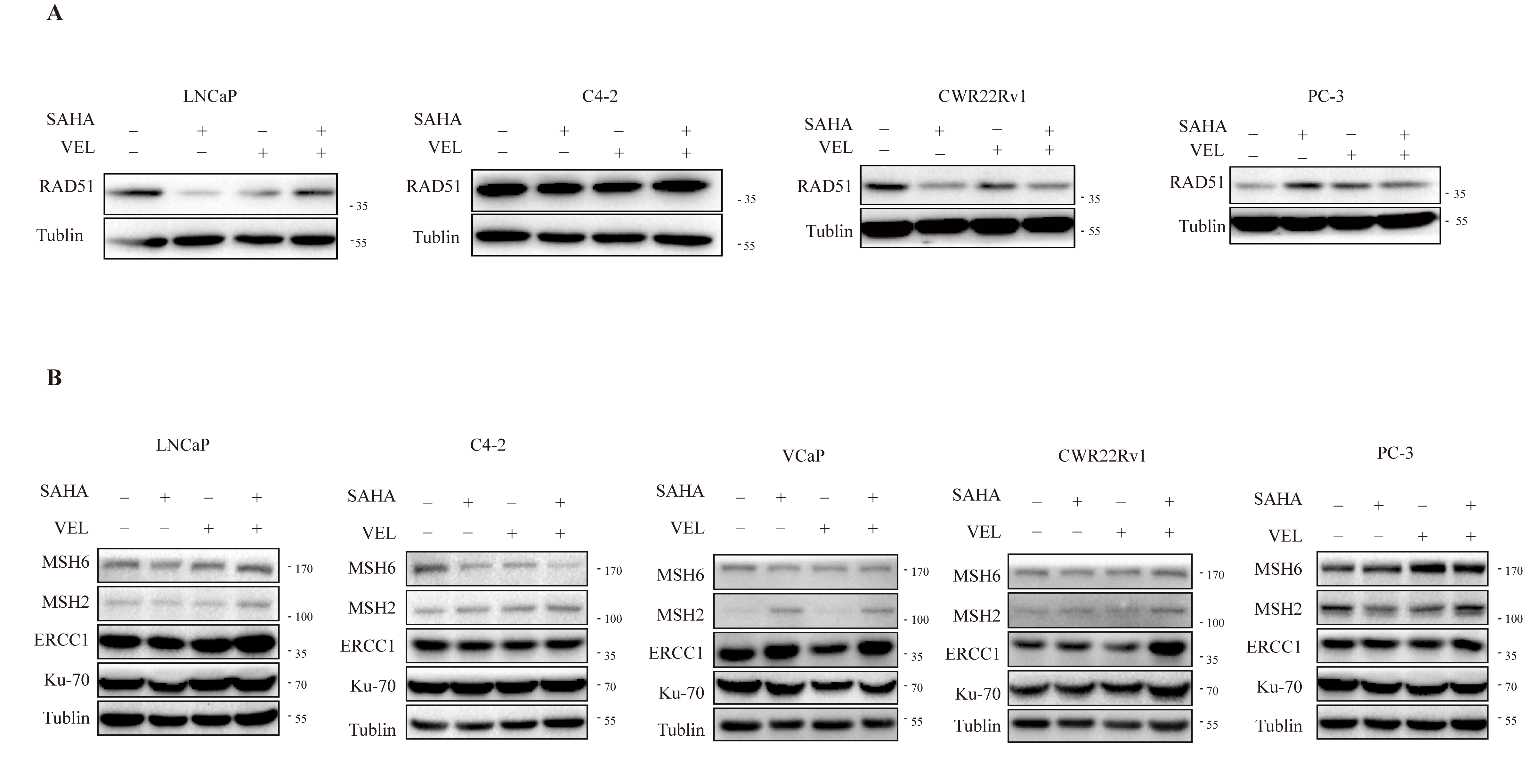

Supplement: Supplementary file 3 — Figure S2. (A) LNCaP, PC-3, CWR22Rv1 and C4–2 cells were treated with SAHA and veliparib alone or in combination for 3 days. The protein levels of RAD51 were assessed by western blot. (B) LNCaP, C4–2, VCaP, CWR22Rv1 and PC-3 cells were treated with SAHA and veliparib alone or in combination for 3 days. The protein levels of DNA damage repair molecules (Ku-70, ERCC1,MSH2 and MSH6) were assessed by western blot. (TIF 2690 kb) [file 13046_2018_810_MOESM3_ESM.tif]
